# Supplementary material for: Complex‐centric proteome profiling by SEC‐SWATH‐MS
Source: Mol Syst Biol. 2019 Jan 14;15(1):e8438. doi: 10.15252/msb.20188438 (PMC6346213; doi:10.15252/msb.20188438)

mTORC2 complex (mTOR/FRAP1, LST8, mAVO3/RICTOR, SIN1)
Annotated subunits: 4   Subunits with signal: 2
Max. coeluting subunits: 2   Max. completeness: 0.5

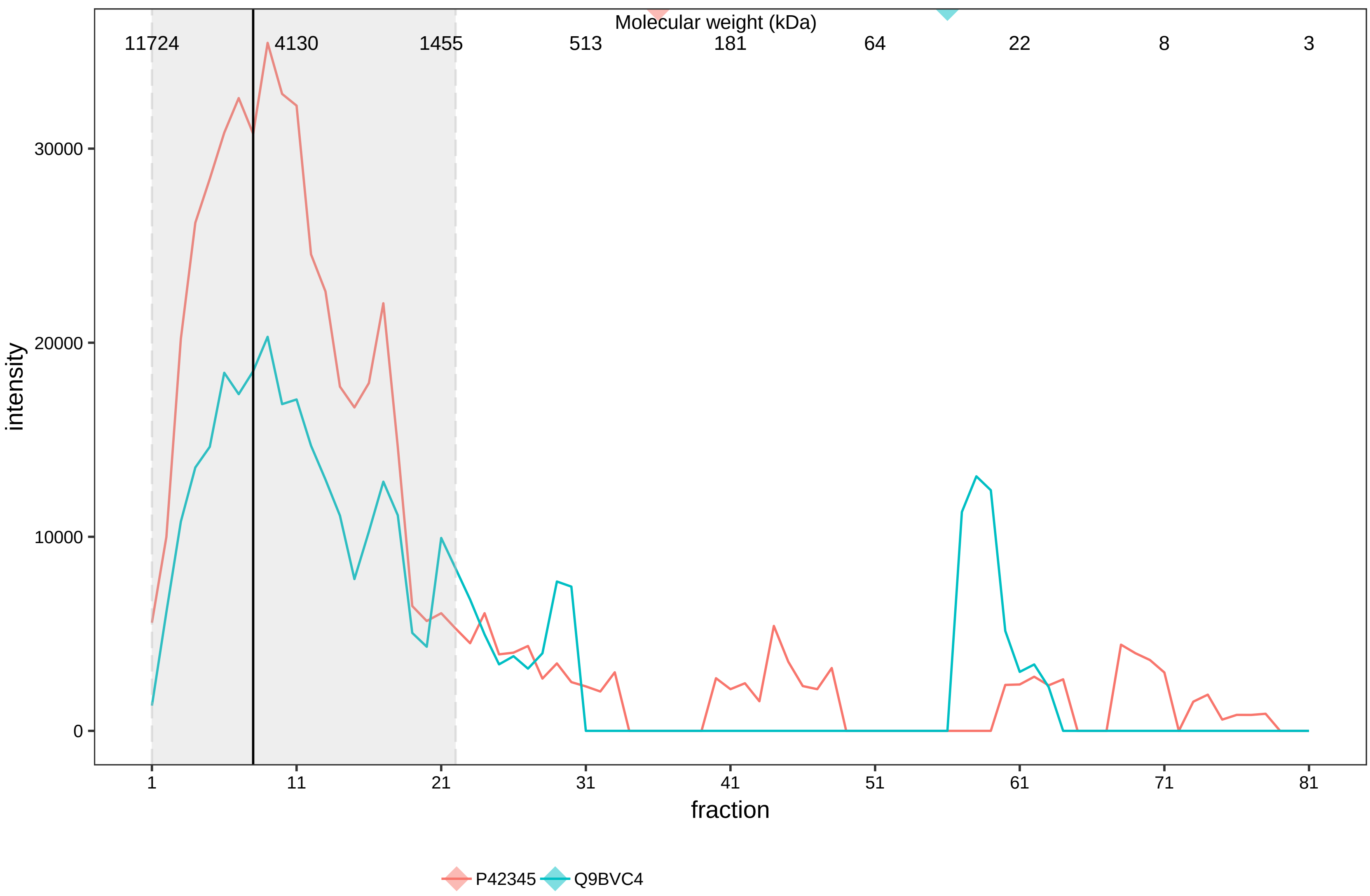

Supplement: Supplementary file 6 — Dataset EV5 [file MSB-15-e8438-s006.zip › feature_plots_corum/3979.pdf]
